# Correlation between pupillary light reflex (PLR) amplitude and visual function in primary angle-closure glaucoma: a retrospective clinical study

**DOI:** 10.1186/s12886-025-04453-6

**Published:** 2025-11-07

**Authors:** Weijia Li, Yulei Geng, Kuitang Shi, Guangxian Tang, Xiaowei Yan, Yawen Li, Tianyu Zhang, Jiaming Lu, Hengli Zhang

**Affiliations:** Shijiazhuang People’s Hospital, Shijiazhuang, China

**Keywords:** Primary angle-closure glaucoma, Mean deviation, Pupillary light reflex, Retinal nerve fiber layer thickness

## Abstract

**Purpose:**

This study investigated the correlation between pupillary light reflex (PLR) amplitude and visual impairment severity in primary angle-closure glaucoma (PACG).

**Methods:**

This retrospective case-control study included 35 eyes of 35 PACG patients and 15 eyes of 15 age-/sex-matched controls. Pupillary parameters (photopic/scotopic diameters, PLR amplitude) were measured using the OPD-Scan III wavefront aberrometer. Clinical metrics included best-corrected visual acuity (BCVA), the cup-to-disc ratio (C/D), the visual field mean deviation (MD), and retinal nerve fiber layer thickness (RNFLT) across six optic disc quadrants. Patients were stratified into mild (MD ≥ -6 dB), moderate (-12 dB ≤ MD < -6 dB), and severe (MD < -12 dB) subgroups per HPA criteria.

**Results:**

Compared to controls, PACG patients showed significantly reduced PLR amplitude and scotopic diameter (*P* < 0.001 and *P* = 0.013, respectively). Significant inter-subgroup differences were observed in PLR amplitude (mild vs. severe; moderate vs. severe) (all *P* < 0.001). Significant inter-subgroup differences were observed in MD (mild vs. severe: *P* < 0.001; moderate vs. mild: *P* = 0.010;moderate vs. severe: *P* = 0.032), BCVA (mild vs. severe: *P* = 0.005), C/D (mild vs. severe: *P* < 0.001; moderate vs. severe: *P* = 0.013), and average RNFLT (mild vs. severe: *P* < 0.001; moderate vs. severe: *P* = 0.032). PLR amplitude negatively correlated with C/D (*r*=-0.706, *P* < 0.001) and positively with MD (*r* = 0.746), nasal (*r* = 0.527), superonasal (*r* = 0.449), inferonasal (*r* = 0.513),Temporal(*r* = 0.475)inferotemporal(*r* = 0.483),and average RNFLT (*r* = 0.526) (all *P* < 0.05). Multivariable linear regression analysis revealed that only MD was independently associated with PLR amplitude (β = 0.040, *P* < 0.001) after adjusting for age, IOP, BCVA, ACD, and IT750.

**Conclusion:**

Reduced PLR amplitude in PACG correlates with structural and functional deterioration, reflecting disease severity. As a rapid, non-invasive metric, PLR assessment complements conventional methods for objective PACG evaluation.

## Introduction

Primary angle-closure glaucoma (PACG) is one of the leading causes of irreversible blindness in China [1–3]. Its pathology is characterized by angle closure-induced elevation in intraocular pressure (IOP), which drives progressive optic neuropathy marked by retinal ganglion cell (RGC) apoptosis, thinning of the retinal nerve fiber layer (RNFL), and characteristic visual field defects [4]. Despite advancements in diagnostic technologies, objective biomarkers for studying disease severity are limited. Current clinical evaluations predominantly rely on structural or functional endpoints, including IOP measurement, optic disc assessment, visual field testing, and optical coherence tomography (OCT). However, these methods may exhibit inherent variability or depend heavily on patient cooperation. Emerging evidence highlights the pupillary light reflex (PLR), a quantifiable neurophysiological response reflecting retinal and optic nerve afferent pathway integrity, as a promising objective biomarker. PLR abnormalities, including attenuated constriction amplitude, prolonged latency, and reduced constriction velocity, are closely associated with the severity of glaucomatous optic nerve damage [5, 6]. Notably, these PLR alterations are more pronounced in glaucoma patients than in healthy controls [7–9]. Nevertheless, existing studies have focused predominantly on primary open-angle glaucoma (POAG) [10], while systematic investigations into PLR characteristics in the PACG—a distinct entity with unique pathophysiological mechanisms—remain scarce. The role of the PLR in PACG severity has yet to be fully elucidated. To address this gap, this study aimed to investigate the clinical characteristics of the PLR amplitude in PACG patients and its correlation with established visual function metrics, including best-corrected visual acuity (BCVA), the cup-to-disc ratio (C/D), visual field mean deviation (MD), and sectoral RNFL thickness. By validating the PLR as an objective, noninvasive biomarker for tracking PACG severity, our findings seek to refine clinical monitoring strategies and inform early intervention in this sight-threatening disorder.

## Materials and methods

### Study participants

This retrospective case‒control study included 35 PACG patients (70 eyes) diagnosed at the Ophthalmology Department of Shijiazhuang People’s Hospital from January 1, 2023, to August 1, 2024 (PACG group) and 15 age- and sex-matched healthy volunteers (30 eyes) as controls. This study has been approved by the Medical Research Ethics Committee of Shijiazhuang People’s Hospital (No.2025-058). During the treatment consultation, comprehensive information was provided to all patients and their families, who voluntarily signed informed consent forms related to the treatment.The research complied with the Declaration of Helsinki.

#### Inclusion criteria

The PACG groups were as follows: (1) diagnosed with PACG according to the Chinese glaucoma guidelines (2020 Edition) [11]; (2) aged ≥ 40 years; (3) diagnosed with glaucomatous optic nerve damage and characteristic visual field defects.

Control group: (1) Intraocular pressure (IOP) < 21 mmHg (1 mmHg = 0.133 kPa); (2) normal optic disc morphology (ISNT rule) and mean deviation (MD) of visual field within the normal reference range (-2 dB < MD < + 2 dB); (3) no history of ocular diseases or family history of glaucoma.

#### Exclusion criteria

(1) use of topical or systemic medications affecting pupil movement, such as pilocarpine and antidepressants; (2) postacute attack mydriasis (sluggish or absent light reflex); (3) comorbid pathological myopia, amblyopia, or other retinal/optic neuropathies; (4) history of ocular trauma or intraocular surgery; (5) nuclear sclerosis greater than grade 2 as assessed by slit-lamp examination; (6) secondary glaucoma (e.g., pseudoexfoliation syndrome, pigment dispersion syndrome); (7) active uveitis or local factors (e.g., iris adhesion, pupillary deformity) affecting pupillary function; (8) systemic diseases such as diabetes, thyroid disorders, or neurologic diseases.

PACG patients were classified into three subgroups on the basis of the Hodapp-Parrish-Anderson (HPA) staging criteria [12, 13]: mild (MD ≥-6 dB), moderate (-12 dB ≤ MD < -6 dB), and severe (MD < -12 dB).

### Study procedures

#### Routine examinations

All participants underwent standardized ophthalmic examinations, including BCVA measurement (converted to LogMAR values) via the international standard visual acuity chart, anterior segment evaluation via slit-lamp microscopy, IOP measurement with Goldmann applanation tonometry, gonioscopy, and fundus examination.

#### Measurement parameters

Visual field MD was assessed via a Humphrey Field Analyzer (30 − 2 SITA standard program; Carl Zeiss, Germany). The retinal nerve fiber layer thickness (RNFLT) in six optic disc quadrants (nasal, superonasal, inferonasal, temporal, superotemporal, inferotemporal) and the average RNFLT thickness were measured via optical coherence tomography (OCT; Heidelberg Engineering, Germany). The cup-to-disc ratio (C/D) was determined via direct ophthalmoscopy. Iris thickness at 750 μm from the scleral spur (IT750) and anterior chamber depth (ACD) was measured using ultrasound biomicroscopy (UBM; MD-300 W, China).

#### Pupilometry parameter measurement

**Fig. 1:**
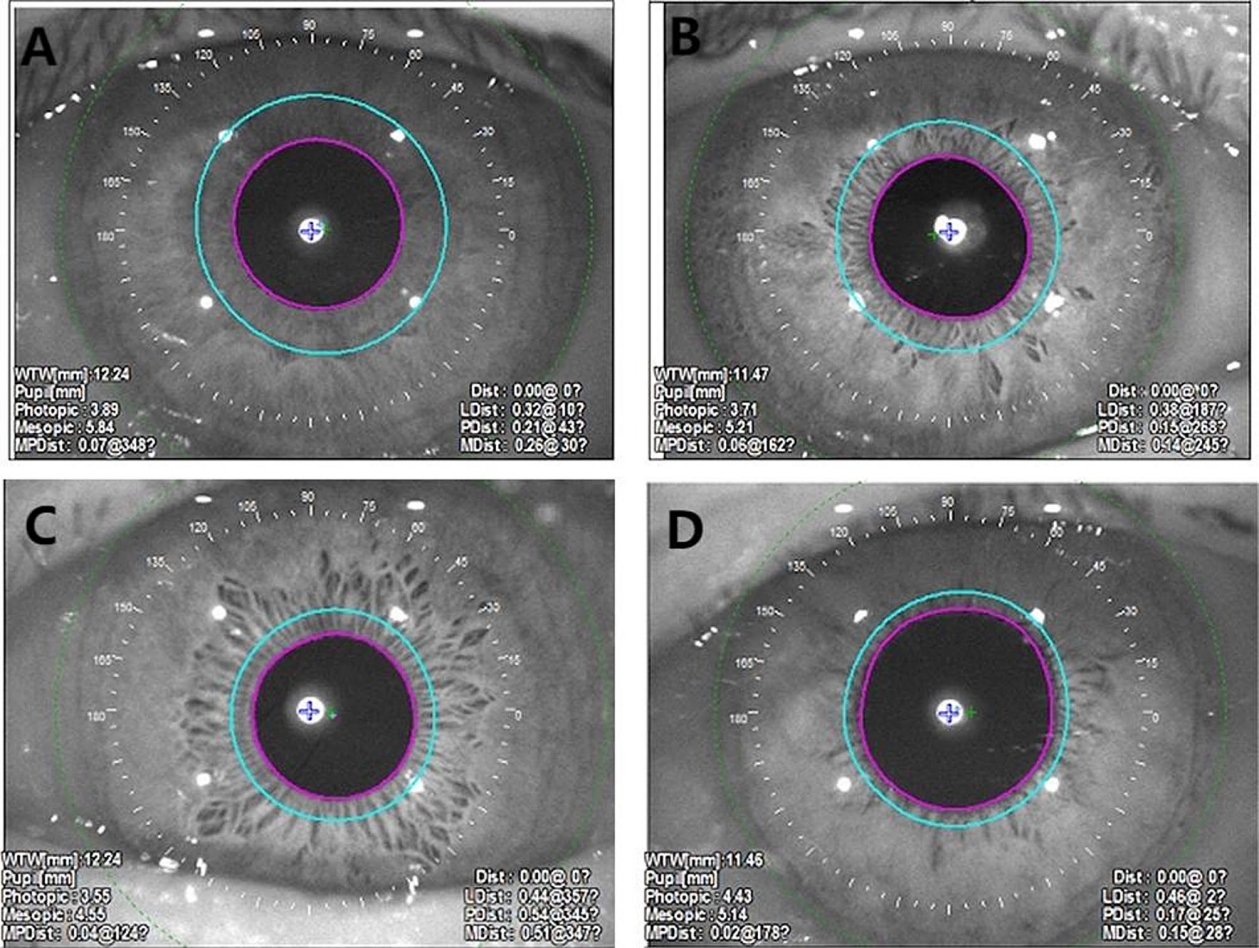
Pupillometric imaging using OPD-SCAN III in study participants. (**A**) Control group: Photopic pupil diameter: 3.89 mm; scotopic pupil diameter: 5.84 mm; PLR amplitude: 1.98 mm. (**B**) Mild PACG group: Photopic pupil diameter: 3.71 mm; scotopic pupil diameter: 5.21 mm; PLR amplitude: 1.50 mm. (**C**) Moderate PACG group: Photopic pupil diameter: 3.55 mm; scotopic pupil diameter: 4.55 mm; PLR amplitude: 1.00 mm. (**D**) Severe PACG group: Photopic pupil diameter: 4.43 mm; scotopic pupil diameter: 5.14 mm; PLR amplitude: 0.71 mm. Note: Device-labeled ‘mesopic’ diameter represents scotopic-equivalent measurement per McDonnell et al. (2006) [15]

Pupil parameters were measured using the OPD-Scan III wavefront aberrometer (NIDEK, Japan). This integrated device combines pupillometry, corneal topography, and wavefront aberration analysis, with its measurement validity for pupillometry clinically established in prior studies [14]. All the measurements followed standardized protocols to ensure consistency. The operational procedure was as follows: Participants underwent 20 min of dark adaptation in a light-controlled room (ambient illumination < 1 lx, verified by a calibrated LX-1330B lux meter). seated subjects stabilized their heads via a chin rest and forehead support. The clinician aligned the pupil center with the optical axis of the device via adjustable hand control, triggered image capture. Measurements affected by blinks, poor focus, or inadequate pupil exposure were discarded and immediately repeated. The mean value of three valid measurements per condition (scotopic/photopic) was used for analysis. (Fig. 1 the pupil parameters of the subjects were measured via OPD-Scan III).

##### PLR amplitude calculation

PLR amplitude was defined as the absolute difference between scotopic and photopic pupil diameters:

$$\:PLR\:amplitude\:\left(mm\right)\hspace{0.17em}=\hspace{0.17em}scotopic\:pupil\:diameter\hspace{0.17em}-\hspace{0.17em}photopic\:pupil\:diameter$$

### Statistical analysis

The data were analyzed via SPSS 27.0 and GraphPad Prism. To account for the inclusion of both eyes from some participants and to adhere to the assumption of statistical independence, one eye per subject was randomly selected for all statistical analyses. The normality of continuous variables was assessed via the Shapiro‒Wilk test. Normally distributed data are expressed as the mean ± standard deviation (SD) and were compared via independent t tests. Nonnormally distributed data are presented as medians (Q1, Q3) and were analyzed with Wilcoxon rank-sum tests for between-group comparisons, Kruskal‒Wallis H tests for multigroup comparisons, and Bonferroni correction for pairwise comparisons. Spearman rank correlation analysis was used to evaluate the associations between pupillary parameters and BCVA, IT750, MD, C/D, and RNFLT.A multivariable linear regression model was employed to identify factors independently associated with PLR amplitude.

## Results

### Demographic characteristics

After random selection of one eye per subject, the analysis included 35 eyes from 35 PACG patients (15 males, 20 females), and 15 eyes from 15 healthy controls (5 males, 10 females). No significant differences were observed between the groups in terms of age (t = 0.051, *P* = 0.959) or sex distribution (χ² = 1.823, *P* = 0.187) (Table 1).

### Intergroup comparison of pupillary parameters

Significant differences in scotopic pupil diameter and PLR amplitude were found between the PACG and control groups(*P* < 0.05). No significant differences were observed in the photopic pupil diameter (*P* > 0.05) (Table 2).

### Subgroup analysis of PACG patients

Based on the HPA stage, PACG patients were divided into mild (15 eyes), moderate (8 eyes), and severe (12 eyes) subgroups. No significant differences were observed among the subgroups in terms of sex (χ² = 1.130, *P* = 0.659) or age (H = 1.975, *P* = 0.372).

#### Ocular parameters across PACG subgroups

Significant differences were observed among subgroups in MD (*P* < 0.001). Post hoc analysis revealed differences between the mild and severe subgroups (*P* < 0.001), between the mild and moderate subgroups (*P* = 0.010),between the moderate and severe subgroups (*P* = 0.032). BCVA differed significantly across subgroups (*P* = 0.016), with mild vs. severe subgroups showing a difference (*P* = 0.005). The C/D ratios also varied significantly (*P* < 0.001), with differences between the mild and severe subgroups (*P* < 0.001) and between the moderate and severe subgroups (*P* = 0.013). Significant differences were observed among subgroups in average RNFLT(*P* = 0.003). Post hoc analysis revealed differences between the mild and severe subgroups (*P* < 0.001) and between the moderate and severe subgroups (*P* = 0.032) (*P* > 0.05) (Table 3).

#### Pupillary parameters across PACG subgroups

PLR amplitude differed significantly among subgroups (*P* < 0.001), with a notable decrease in the severe subgroup compared with the mild subgroup and the moderate subgroup (*P* < 0.001). No significant differences were observed in the photopic or scotopic pupil diameter (*P* > 0.05) (Table 3).

### Correlation analysis of pupillary parameters

PLR amplitude was negatively correlated with C/D (*r* = -0.706, *P* < 0.001) and positively correlated with MD (*r* = 0.746), nasal (*r* = 0.527), superonasal (*r* = 0.449), inferonasal (*r* = 0.513), Temporal(*r* = 0.475), inferotemporal (*r* = 0.483), and average RNFLT (*r* = 0.526) (all *P* < 0.05). Photopic pupil diameter was positively correlated with BCVA (*r* = 0.352, *P* = 0.038). The scotopic pupil diameter was negatively correlated with ACD (*r* = -0.342, *P* = 0.044) (Table 4).

### Simple linear regression analyses between PLR amplitude and key parameters

**Fig. 2:**
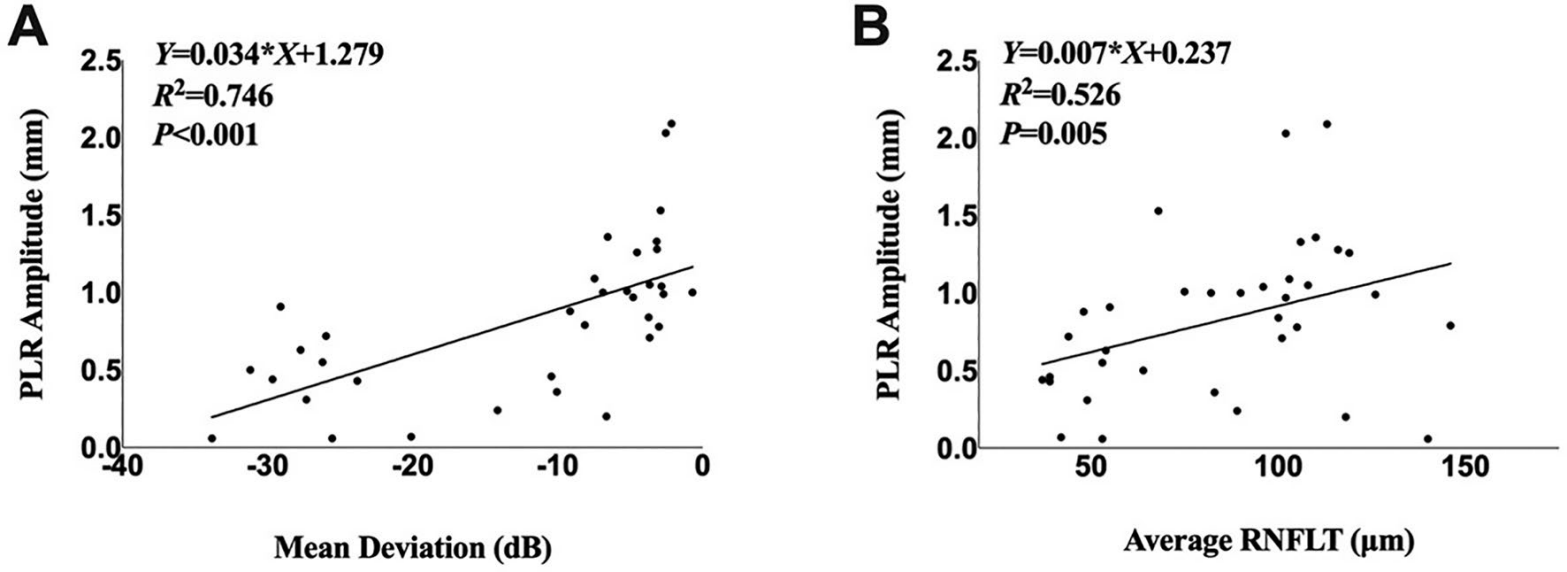
Scatterplots showing correlations between PLR amplitude and (**A**) MD and (**B**) average RNFLT. (**A**) Scatterplot showing a significant positive correlation between MD and PLR amplitude, (R² = 0.746, *P* < 0.001). (**B**) Scatterplot showing a significant positive correlation between average RNFLT and PLR amplitude, (R² = 0.526, *P* = 0.005)

A strong positive correlation was observed between PLR amplitude and MD. For every 1 dB decrease in MD, the PLR amplitude decreased by 0.034 mm (*P* < 0.001). Similarly, a significant positive correlation was found between PLR amplitude and average RNFLT. For every 1 μm decrease in average RNFL thickness, the PLR amplitude decreased by 0.007 mm (*P* = 0.005)(Fig. 2).

### Results of multivariable linear regression analysis

Multivariable linear regression analysis revealed that only MD was independently associated with PLR amplitude (β = 0.040, *P* < 0.001) after adjusting for age, IOP, BCVA, ACD, and IT750.The overall model was statistically significant and explained 49.5% of the variance in PLR amplitude (Table 5).

**Table 1:** Demographic and ocular parameter comparisons between the PACG group and the control group

Parameter	PACG (*n* = 35)	Normal (*n* = 15)	*p* value
Age	63.9 ± 8.2	63.8 ± 7.6	0.959
**Sex**,** n (%)**			0.187
Male	15(42.8)	5(33.3)	
Female	20(57.2)	10(66.7)	
*BCVA* (LogMAR)	0.23 (0.13,0.39)	0.22(0.00,0.30)	0.219
IOP (mmHg)	18.50 (16.42,20.76)	16.00(12.00,17.00)	<0.001
MD (dB)	-6.87(-25.11, -3.24)	0.22(-0.78,0.97)	<0.001
ACD (mm)	1.92 (1.77,2.07)	2.69(2.45,2.82)	<0.001
IT750 (mm)	0.48 (0.45,0.51)	0.39(0.38,0.45)	0.002
Endothelial cell density(mm^2^)	2606.00 (2252.75,2771.75)	2877.00(2405.00,3014.00)	0.058
Superonasal RNFLT (um)	91.11 ± 36.17	113.86 ± 21.40	0.008
Nasal RNFLT (um)	64.23 ± 21.95	74.46 ± 16.15	0.111
Inferonasal RNFLT (um)	96.06 ± 40.62	120.67 ± 18.64	0.005
Superiotemporal RNFLT (um)	109.40 ± 47.10	134.80 ± 20.76	0.011
Temporal RNFLT (um)	66.37 ± 20.29	78.40 ± 13.69	0.042
Inferotemporal RNFLT (um)	112.00 ± 51.13	149.87 ± 20.05	<0.001
Average RNFLT (um)	85.00 ± 31.36	101.40 ± 7.05	0.006

**Table 2:** Comparison of pupil parameters between the PACG group and the control group

Parameter	PACG (*n* = 35)	Normal (*n* = 15)	*p* value
Photopic pupil diameter (mm)	3.53(2.98,4.45)	3.33(3.04,3.80)	0.368
Scotopic pupil diameter (mm)	4.40(3.86,5.23)	4.73(4.50,5.47)	0.013
PLR Amplitude (mm)	0.88(0.47,1.17)	1.49(1.35,1.66)	<0.001

**Table 3:** Comparison of PACG subgroups

Parameter	Mild (*n* = 15)	Moderate (*n* = 8)	Severe (*n* = 12)	*p* value
MD (dB)	-3.15(-3.73, -2.71)	-7.79(-9.83, -6.69)	-26.77(-29.51, -24.23)	< 0.001
Average RNFLT (um)	102.00(96.00,113.00)	96.50(56.75,116.00)	53.00(42.50,61.75)	0.003
BCVA	0.22(0.10,0.22)	0.22(0.10,0.28)	0.46(0.22,1.00)	0.016
C/D	0.40(0.30,0.40)	0.45(0.33,0.88)	1.0(1.0,1.0)	< 0.001
IT750 (mm)	0.49(0.47,0.50)	0.46(0.44,0.52)	0.46(0.44,0.54)	0.294
Photopic pupil diameter (mm)	3.39 (2.80,3.90)	3.29 (2.97,4.98)	4.00 (3.21,5.90)	0.156
Scotopic pupil diameter (mm)	4.55 (3.85,5.14)	4.37 (3.76,5.22)	4.44 (3.63,6.35)	0.871
PLR Amplitud (mm)	1.04 ± 0.41	1.01 ± 0.30	0.36 ± 0.22	< 0.001

**Table 4:** Correlation analysis between pupil parameters and visual function indicators in PACG patients

Parameter	PLR Amplitude	photopic pupil diameter	scotopic pupil diameter
BCVA	-0.177	0.352*	0.317
ACD	-0.021	0.318	-0.342*
IT750	0.082	-0.233	-0.214
MD	0.746***	-0.299	-0.043
C/D	-0.706***	0.318	0.072
Nasal RNFLT	0.527***	-0.015	0.165
Superonasal RNFLT	0.449**	-0.026	0.170
Inferonasal RNFLT	0.513**	-0.055	0.120
Temporal RNFLT	0.475**	0.170	0.321
Superiotemporal RNFLT	0.483**	-0.031	0.182
Inferotemporal RNFLT	0.546***	0.075	0.234
Average RNFLT	0.526**	-0.035	0.157

All values are Spearman correlation coefficients (r). Statistical significance is denoted as *p < 0.05, **p < 0.01, and ***p < 0.001

**Table 5:** Multivariable linear regression analysis for factors associated with PLR amplitude

Parameter	Unstandardized Coefficient (B)	Standard Error	Standardized Coefficient (Beta)	t	*p* value
Constant	1.115	0.867		1.286	0.209
Age	-0.001	0.008	-0.013	-0.102	0.920
IOP	0.003	0.007	0.063	0.466	0.645
BCVA	0.385	0.258	0.230	1.492	0.147
ACD	0.023	0.245	0.013	0.095	0.925
IT750	0.115	1.060	0.014	0.108	0.914
MD	0.040	0.007	0.884	5.765	< 0.001

Model Summary: R² = 0.584, Adjusted R² = 0.495, F-statistic = 6.551, p-value for the model = < 0.001

## Discussion

PACG, a complex disease with a high prevalence in Asia [2, 16], has been investigated predominantly through invasive and technically demanding methods such as molecular genetics and metabolomics for biomarker discovery. In contrast, PLR assessment—simple, noninvasive, and rapid—has emerged as a promising frontier. This study systematically evaluated PLR characteristics in PACG patients, revealing correlations with visual functional impairment and providing novel objective evidence for evaluating glaucoma severity.

Our findings demonstrated significantly reduced PLR amplitude in the PACG group compared with the control group, which aligns with the findings of Hao et al. [17] of slower iris contraction velocity and reduced acceleration in PACG using dynamic iris imaging. Furthermore, we confirmed that IT750 was significantly greater in PACG eyes than in controls. However, no significant correlation was observed between IT750 and PLR amplitude, indicating that iris thickening, although characteristic of PACG, is not a major contributing factor to the reduced PLR. This conclusion is further strengthened by our multivariable regression model, which identified MD as the only independent factor predicting PLR amplitude, while ACD and IT750 did not show significant associations.

Using HPA staging, we observed a progressive decline in the PLR amplitude with disease severity, which is consistent with the findings of Martucci et al. [18], who applied the GSS2 staging system. Moreover, the PLR amplitude was positively correlated with the MD and average RNFLT and negatively correlated with the C/D. These findings are in agreement with previous studies: Rao et al. [19] reported that intereye differences in PLR amplitude predict RNFLT asymmetry, while Pradhan et al. [20] and Najjar et al. [10] reported significant associations between PLR parameters and RNFLT, reflecting optic nerve damage. Histological studies have confirmed that advanced glaucoma involves a reduced density of intrinsically photosensitive retinal ganglion cells (ipRGCs) [21], which directly impairs the PLR amplitude [7, 22]. Pathologically, reduced PLR amplitude may be associated with ipRGC dysfunction, as supported by histological evidence of ipRGC depletion in glaucoma [7, 21, 23]^,^.

Notably, although a diminished PLR amplitude is a recognized feature of glaucomatous optic neuropathy, our findings suggest that the mechanism in PACG also involves other factors. The observed restriction in scotopic pupil diameter implies that iris constraints limit pupillary mobility, adding a unique component to the PLR pathology in PACG that distinguishes it from the primarily neurogenic deficit in POAG [24–26]. Future studies directly comparing pupillometric parameters between PACG and POAG cohorts are warranted to further elucidate these differential mechanisms.

This study utilized OPD-Scan III for pupillometry, a device validated for high accuracy and reproducibility in low-light conditions [14, 15, 27]. However, several limitations exist: (1) the small sample size may reduce statistical power; (2) the retrospective design precludes causal inference, warranting future multicenter cohort studies; (3) subjective visual field testing introduces potential bias, necessitating complementary objective functional assessments; (4)This cross-sectional design does not allow us to evaluate whether PLR parameters can serve as a reliable marker of future disease progression, future longitudinal studies are warranted.

## Conclusion

Our findings demonstrate that a reduced PLR amplitude in patients with PACG is significantly associated with the severity of visual functional impairment. Furthermore, PLR assessment serves as a valuable complement to conventional methods for objective evaluation of PACG severity.

## Data Availability

The datasets utilized and analyzed during the current study are available from the corresponding author upon reasonable request.

## Abbreviations

PACG: Primary Angle-Closure Glaucoma
PLR: Pupillary Light Reflex
MD: Mean Deviation
RNFLT: Retinal Nerve Fiber Layer Thickness
C/D: Cup-to-Disc Ratio
ipRGCs: intrinsically photosensitive Retinal Ganglion Cells
UBM: Ultrasound Biomicroscopy
IT750: Iris Thickness at 750 μm
HPA: Hodapp-Parrish-Anderson
POAG: Primary Open-Angle Glaucoma
